# Positron Emission Tomography/Computed Tomography Imaging‐Guided Polydopamine Nanoparticles Attenuate Foam Cell Ferroptosis for Targeted Antiatherosclerotic Therapy

**DOI:** 10.1002/smsc.202500221

**Published:** 2025-07-06

**Authors:** Ximei Dai, Zhiyue Wang, Jiaqi Lu, Yutong Xu, Xingji Liu, Jianchen Qi, Tao Zheng, Feng Wang, Guangming Lu, Longjiang Zhang, Jie Sheng, Guifen Yang

**Affiliations:** ^1^ Department of Nuclear Medicine Jinling Clinical Medical College Nanjing University of Chinese Medicine Nanjing Jiangsu 210008 China; ^2^ Department of Radiology Jinling Hospital, Affiliated Hospital of Medical School Nanjing University Nanjing Jiangsu 210008 China; ^3^ Department of Radiology Jinling Clinical Medical College Nanjing University of Chinese Medicine Nanjing Jiangsu 210008 China; ^4^ Department of Nuclear Medicine Nanjing First Hospital Nanjing Medical University Nanjing Jiangsu 210008 China; ^5^ Department of Radiology Jinling Hospital The First School of Clinical Medicine Southern Medical University Nanjing Jiangsu 210008 China; ^6^ Department of Nuclear Medicine Jinling Hospital Affiliated Hospital of Medical School Nanjing University Nanjing Jiangsu 210008 China

**Keywords:** atherosclerosis, ferroptosis, positron emission tomography/computed tomography, polydopamine, theranostics

## Abstract

Atherosclerosis (AS) is a significant contributor to cardiovascular events. Recent studies have demonstrated that ferroptosis of foam cells is a significant driver of AS. Nevertheless, insights into the precise antiferroptosis therapies remain limited. Here, a multifunctional theranostic nanoplatform is engineered by conjugating folate‐modified polydopamine (PDA) nanoparticles (NPs) with L‐arginine (FPLG) to inhibit ferroptosis of foam cells. In vitro studies demonstrate that FPLG NPs effectively attenuate ferroptosis in oxidized low‐density lipoprotein (ox‐LDL)‐stimulated macrophages by scavenging reactive oxygen species, upregulating GPX4 and NRF2 activity, and regulating lipid metabolism. In vivo, FPLG NPs exhibit preferential accumulation in atherosclerotic plaques via folate receptors (FRs)‐mediated targeting and the enhanced permeability and retention effect (EPR effect), as confirmed by fluorescence imaging and chelator‐free ^68^Ga‐labeled positron emission tomography/computed tomography (PET/CT). Treatment with FPLG NPs in ApoE^−/−^ mice reduces plaque area by over 40%, enhances fibrous cap stability, and mitigates ferroptosis. Transcriptomics further reveals that the FPLG treatment suppresses ferroptosis and inflammatory pathways. This dual‐modality platform integrates targeted ferroptosis inhibition and real‐time imaging, offering a promising strategy for precise AS management.

## Graphical abstract

Folate‐ and arginine‐modified polydopamine nanoparticles (FPLG NPs) are designed for the specific targeting of atherosclerotic foam cells. These nanoparticles combat ferroptosis by scavenging reactive oxygen species, regulating iron/lipid metabolism, and upregulating the GPX4/NRF2 pathway. Using chelator‐free ^68^Ga‐labeling, positron emission tomography/computed tomography imaging confirms plaque accumulation and guides therapeutic intervention. The theranostic FPLG NPs achieves a plaque reduction of over 40%, thereby offering a precise theranostic strategy.

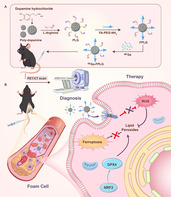

## 1 Introduction

Atherosclerosis (AS), characterized by chronic inflammation and lipid accumulation within arterial walls, remains the leading cause of cardiovascular morbidity and mortality worldwide.^[^ ^1^ ^]^ Despite advancements in lipid‐lowering therapies, residual cardiovascular risks persist, underscoring the urgent need for novel therapeutic strategies targeting alternative pathological pathways.^[^ ^2^ ^]^

Emerging evidence highlights the pivotal role of ferroptosis, an iron‐dependent form of regulated cell death featured by lipid peroxidation and glutathione peroxidase 4 (GPX4) inactivation, in promoting plaque instability and disease progression.^[^ ^3^ ^]^ In atherosclerotic lesions, macrophages engulf oxidized low‐density lipoprotein (ox‐LDL) to form lipid‐laden foam cells, which are particularly susceptible to ferroptosis due to their dysregulated iron metabolism and elevated oxidative stress.^[^ ^4^, ^5^ ^]^ The accumulation of redox‐active iron within these cells catalyzes Fenton reactions, exacerbating lipid peroxidation and overwhelming endogenous antioxidant defenses.^[^ ^6^ ^]^ This process leads to membrane rupture, release of damage‐associated molecular patterns (DAMPs), and propagation of inflammation, ultimately contributing to necrotic core expansion, fibrous cap thinning, and plaque rupture.^[^ ^7^ ^]^ Current interventions targeting ferroptosis, however, face significant limitations, including systemic toxicity, poor bioavailability, and the inability to monitor therapeutic efficacy in real time.^[^ ^8^ ^]^

Polyphenolic materials have garnered numerous attention for their intrinsic antioxidant properties, biocompatibility, metal chelating ability, and versatile surface chemistry.^[^ ^9^ ^]^ Polydopamine nanoparticles exhibit robust free radical scavenging capabilities and can chelate redox‐active iron ions, making them ideal candidates for combating ferroptosis‐related oxidative damage.^[^ ^10^, ^11^ ^]^

Notably, the catechol‐rich structure of PDA enables facile integration of diagnostic functionalities, such as chelator‐free radiolabeling with metal isotopes (such as ^68^Ga), allowing real‐time positron emission tomography/Computed tomography (PET/CT) imaging to track nanoparticle biodistribution and plaque targeting efficiency.^[^ ^12^, ^13^ ^]^ Arginine, a conditionally essential amino acid, synergistically potentiates cellular antioxidant defense systems through molecular interplay with PDA, where its cationic guanidinium group optimizes PDA's free radical scavenging topology while stabilizing redox‐active intermediates.^[^ ^14^, ^15^ ^]^ To achieve targeted delivery, FRs overexpressed on foam cells within atherosclerotic plaques, offer a promising strategy for site‐specific accumulation of therapeutic agents.^[^ ^16^ ^]^

**Scheme 1:**
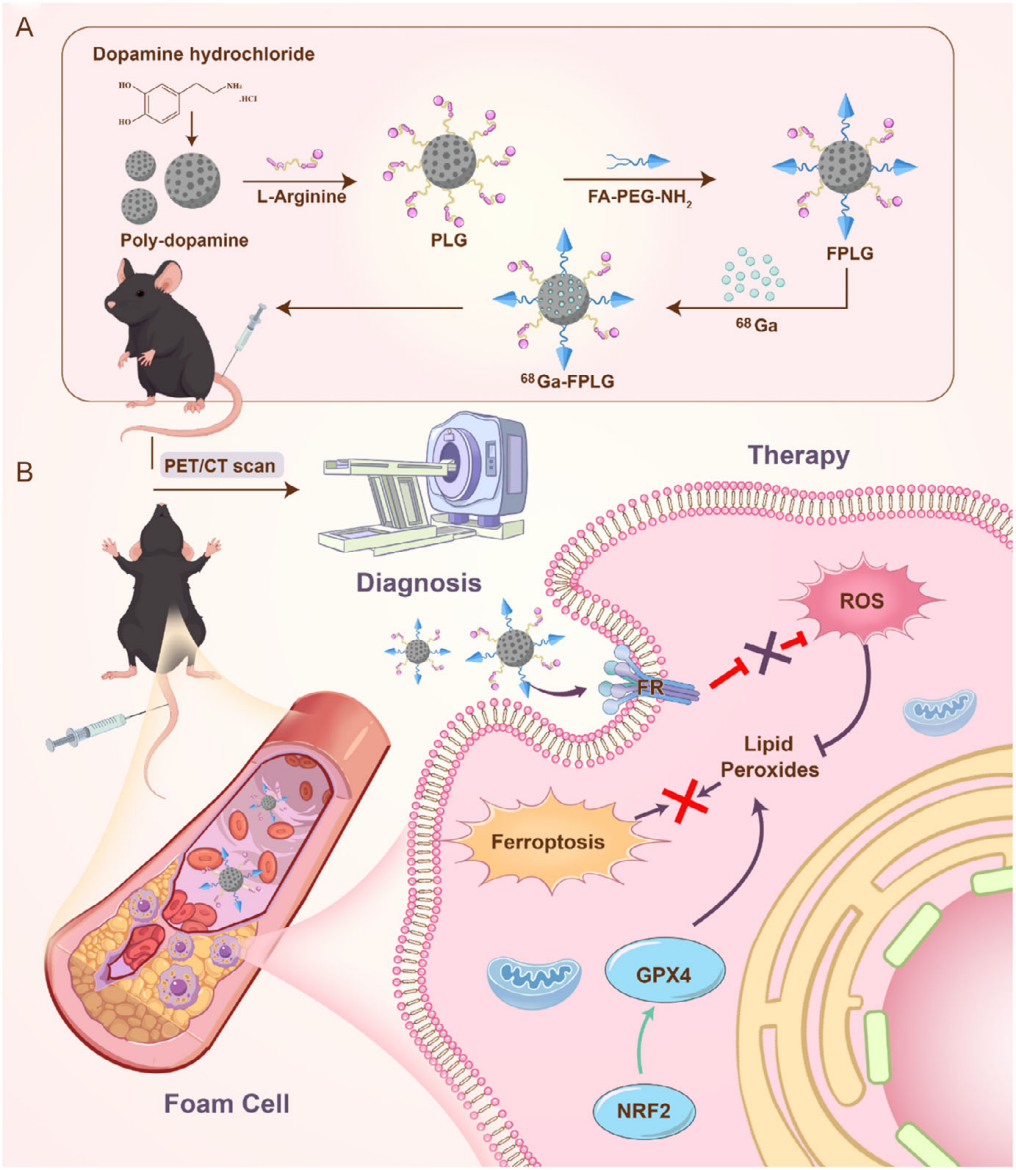
Schematic illustration of FPLG nanoparticles for AS therapy. A) Diagrammatic depiction of the synthesis protocol for surface‐engineered FPLG NPs and subsequent radiolabeling with ^68^Ga for PET/CT imaging. B) Schematic illustration depicting the multifaceted antiatherogenic mechanisms of FPLG NPs, including FR‐mediated targeting of vulnerable atherosclerotic plaques combined with ferroptosis inhibition through ROS scavenging, downregulation of GPX4 and NRF2 expression.

In this study, we engineered a multifunctional theranostic nanoplatform by conjugating folate‐modified PDA NPs with L‐arginine (FPLG) to inhibit the ferroptosis of foam cells. We demonstrated that FPLG NPs effectively attenuated ferroptosis in ox‐LDL‐stimulated macrophages by scavenging the reactive oxygen species (ROS), increasing GPX4 and NRF2 activity, and modulating lipid metabolism. In vivo studies further revealed that FPLG NPs could alleviate AS by reducing lipid accumulation, enhancing fibrous cap thickness, promoting endothelial repair and reducing macrophage infiltration. Additionally, the FPLG NPs could be radiolabeled with ^68^Ga, enabling noninvasive PET/CT imaging capabilities. FPLG NPs showed notable accumulation in the AS plaque, especially in the foam cells, as confirmed by fluorescence imaging. Importantly, the inherent phenolic hydroxyl groups of PDA facilitated chelator‐free labeling with ^68^Ga for real‐time tracking of nanoparticle biodistribution and plaque targeting. PET/CT imaging confirmed the prolonged retention of ^68^Ga‐labeled NPs within atherosclerotic lesions, providing a feasible tool to optimize dosing regimens and validate therapeutic targeting. This dual‐modality approach not only addresses ferroptosis‐driven plaque vulnerability but also bridges the gap between precise treatment and imaging‐guided intervention, offering a paradigm shift in the management of AS (**Scheme** **1**).

## 2 Results and Discussion

### 2.1 Synthesis and Characterization of FPLG NPs

**Figure 1:**
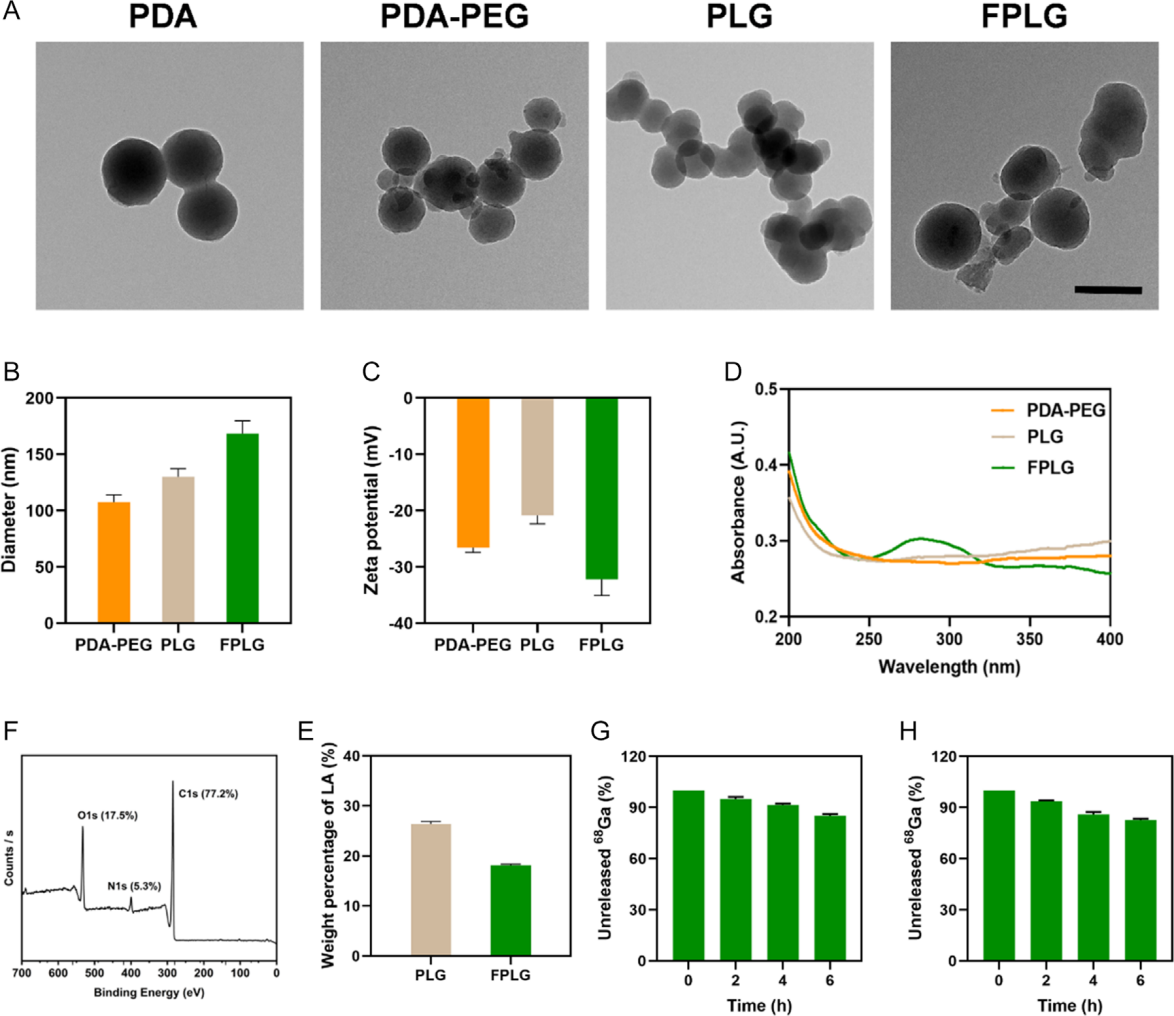
The characterization of the nanoparticles. A) Representative TEM image of PDA, PDA‐PEG, PLG, and FPLG NPs. Scale Bar: 200 nm. B) The hydrodynamic size and C) Zeta potential of PDA‐PEG, PLG, and FPLG NPs. (*n* = 3). D) UV–vis spectrum of PDA‐PEG (100 μg/mL), PLG (50 μg/mL), and FPLG (50 μg/mL). E) XPS survey scan spectra of FPLG NPs. F) Weight percentages of LA in PLG and FPLG NPs (*n* = 3). The radio‐stability of 68Ga labeled FPLGs NPs in G) PBS and H) complete DMEM medium, respectively. Data are expressed as mean ±S.D. (*n* = 3).

The PDA core was prepared through oxidative self‐polymerization methods, as previous studies have reported.^[^ ^17^, ^18^ ^]^ Then the FA‐PEG‐NH_2_ (or NH_2_‐PEG‐NH_2_) and L‐arginine (LA) were attached to PDA NPs via Schiff base and/or Michael addition reactions individually,^[^ ^19^, ^20^ ^]^ yielding products named PDA‐PEG, PLG, and FPLG. TEM image confirmed that the PDA‐based NPs all exhibited spherical shape and excellent dispersion (**Figure** **1**A). Dynamic light scattering analysis displayed that the average sizes of PDA‐PEG, PLG, and FPLG were 107.7 nm, 130.1 nm, and 168.3 nm, respectively (Figure 1B). PDA‐PEG nanoparticles exhibited a zeta potential of ‐26.56 mV, while LA‐functionalized PLG NPs showed a less negative value of ‐20.86 mV, indicative of enhanced cationic character imparted by protonated guanidinium moieties in the arginine ligands.^[^ ^15^, ^21^ ^]^ Subsequent surface modification with folate (FA) induced a remarkable reversal in surface charge characteristics. The FA‐coated FPLG NPs demonstrated a substantial increase in surface negativity, with a zeta potential of ‐32.2 mV (Figure 1C). This pronounced negative shift aligns with established literature reports and likely originates from the exposure of carboxylate groups inherent to the FA molecular structure.^[^ ^22^, ^23^ ^]^ The UV–vis indicated that the PDA‐PEG nanoparticles demonstrated a broad absorption profile spanning 200–500 nm. PLG NPs, upon functionalization with LA, demonstrated a significant enhancement in UV absorption intensity across the examined spectral range, which is in accordance with the previous study.^[^ ^15^ ^]^ Subsequent modification with FA imparted a distinct spectral feature to the nanoparticles (Figure 1D). Specifically, FPLG NPs exhibited a pronounced absorption peak at 283 nm, a wavelength that corresponds to the characteristic absorption of unbound FA molecules.^[^ ^24^ ^]^ All the results displayed above provided systematic optical evidence for the success of nanoparticle functionalization. XPS survey scans identified carbon, nitrogen, and oxygen as primary constituents across all nanoparticle variants (PDA‐PEG, PLG, and FPLG NPs). An obvious increase in nitrogen atomic percentage was observed following LA conjugation (Figure 1E and Figure. S1, Supporting Information), consistent with the incorporation of nitrogen‐rich arginine ligands.^[^ ^15^ ^]^ However, subsequent FA functionalization reduced the nitrogen content, likely due to surface shielding effects from the folate moiety. Quantitative measurements of LA doped in PLG and FPLG were analyzed by the Nihydrin colorimetry, the percentage weights of which were 26.3% and 18.1% (Figure 1F), respectively. Moreover, the FPLG NPs demonstrated robust structural stability by retaining their size in deionized water, Dulbecco's modified eagle medium (DMEM), and phosphate buffer saline (PBS) for up to seven days, as evidenced by the data presented in Figure S2, Supporting Information.

The FPLG NPs can radiolabel the ^68^Ga through phenolic hydroxyl coordination without any chelator. Quantitative assessment of radioactivity efficiency showed that 86 ± 3% of ^68^Ga ions can be combined on the FPLG NPs. The labeling stability of ^68^Ga‐FPLG NPs exceeded 80% in both PBS and complete DMEM medium over 6 h (Figure 1G and Figure 1H), indicating its suitability for micro PET/CT applications.

### 2.2 Cellular Cytotoxicity and Uptake of FPLG NPs

The biocompatibility of these PDA‐oriented nanoparticles was tested on the following cells: RAW 264.7 cells, SMCs and MAECs via cell counting kit‐8 (CCK‐8) assay. As shown in Figure S3, Supporting Information, a 24 h‐incubation with FPLG NPs induced negligible cytotoxicity at concentrations ranging from 0–100 μg mL^−1^. Foam cells within atherosclerotic plaques are the predominant cells that undergo ferroptosis and trigger persistent inflammation in the pathologic microenvironment, so the PDA NPs were designed to specifically target foam cells. In a bid to investigate foam cell internalization of PDA‐PEG, PLGs, and FPLGs, we constructed an in vitro cell model to simulate these lipid‐laden cells using ox‐LDL and Lipopolysaccharide (LPS). The PDA NPs were first labeled with the near‐infrared fluorescent dye IR820, as confirmed by UV–vis spectral analysis (Figure. S4, Supporting Information), followed by 12 h coincubation with foam cells. Flow cytometry analysis and confocal laser microscope both detected the strongest fluorescent signal in the FPLG‐treated group, which was almost fivefold of the control group and was significantly higher than that of PDA‐PEG, PLGs, and FPLGs treated groups (**Figure** **2**A). Quantitative analysis in Figure 2B reveals that LA modification modestly increases cellular uptake by 12.1%, and folic acid modification further improves cellular uptake by 35.6%. In addition, FPLG NPs demonstrated 6.9 t lower cellular uptake in nonlipid‐laden macrophages compared to foam cells (Figure S5, Supporting Information). These results exhibited that surface modification of LA and folic acid onto the PDA‐originated nanoparticles markedly improved their affinity to foam cells, laying the foundation for plaque‐targeted diagnosis and treatment in the subsequent sections.

Figure 2Cellular uptake and in vitro antiferroptosis performance of FPLG NPs. A) Flow cytometry analysis of foam cells incubated with IR‐820 labeled PDA‐PEG, PLG, and FPLG, with B) the according mean fluorescence intensity of each group (*n* = 3). C) Representative confocal fluorescence microscope images showing cellular uptake of IR‐820 labeled PDA‐PEG, PLG, and FPLG. Scale bar: 100 μm. D) Flow cytometry analysis of foam cells stained with DCFH‐DA after varied treatments and E) the quantitative results of the intracellular ROS level (*n* = 3). F) Dil‐ox‐LDL uptake by foam cells and H) BODIPY‐stained intracellular lipid accumulated in foam cells treated with various nanoparticles observed by flow cytometry, with the quantitative analysis in G,I), respectively. J) Typical western blotting bands of NRF2, ACSL4, and GPX4 levels in foam cells under different treatments (*n* = 3). K) Intracellular ROS levels of foam cells stained with DCFH‐DA as detected by the fluorescence microscope. Scale bar: 100 μm. L) Oil Red Oil staining of foam cells under differed treatments. M–O) Quantified data of NRF2, ACSL4, and GPX4 levels in foam cells under different treatments (*n* = 3). P) Iron concentration in foam cells under different treatments (*n* = 3). Scale bar: 100 μm. Data are expressed as mean ± SD. ns, no significance, *p < 0.05; **p < 0.01; ***p < 0.001, and ****p < 0.0001.

**Figure:**
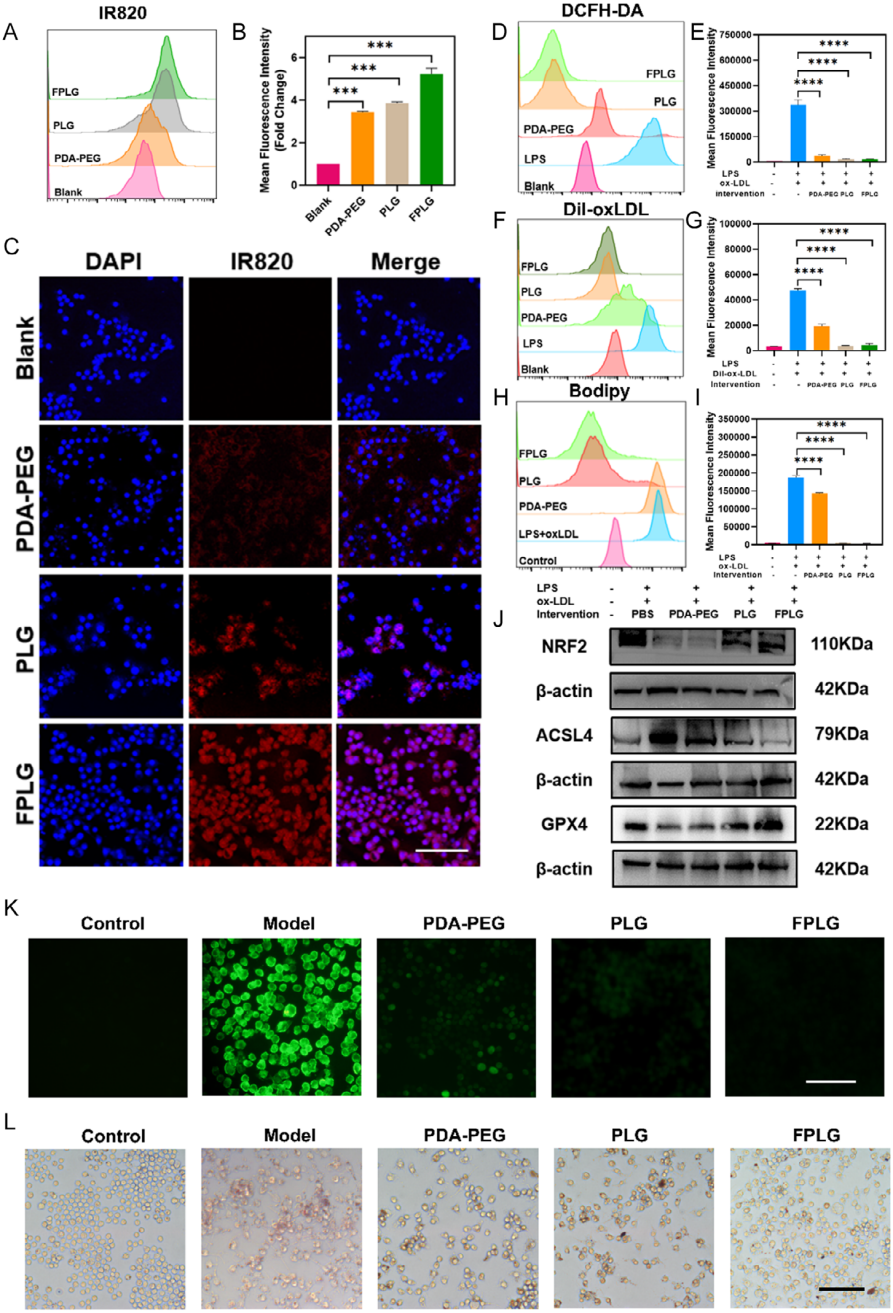

**Figure:**
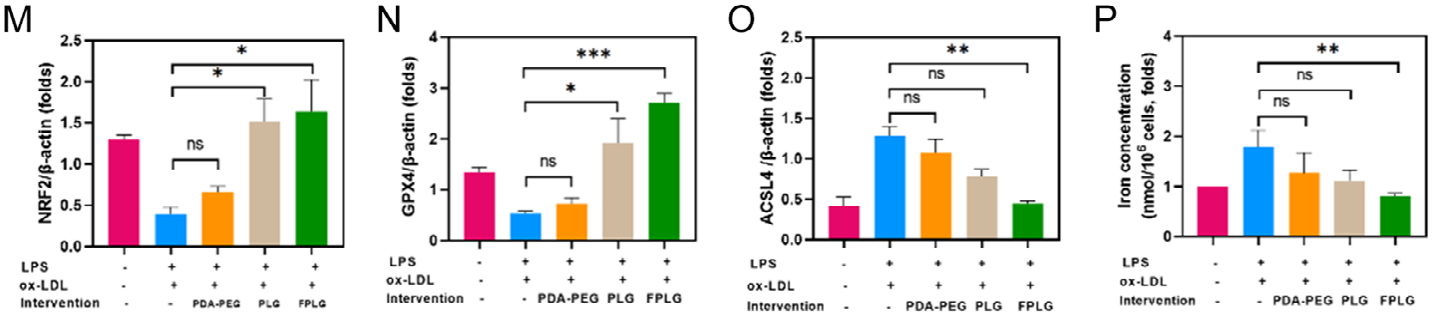

### 2.3 In Vitro Antiferroptosis Performance of FPLG NPs

Ferroptosis is featured by the accumulation of lipid peroxides resulting from compromised antioxidant defense represented by the GPX4‐glutathione system. Excessive ROS in foam cells directly oxidize the polyunsaturated fatty acids in the cell membrane, thereby initiating ferroptosis in the foam cells and then instigating an inflammatory cascade.^[^ ^5^ ^]^ Recent studies show that the employment of antioxidants such as PDA could restore intracellular redox balance and therefore effectively attenuate cell ferroptosis.^[^ ^25^, ^26^ ^]^ As a result, we checked the ROS‐scavenging ability of these PDA‐oriented nanoparticles using the foam cell models constructed in the former section. According to flow cytometry analysis, the fluorescent signal of 2,7‐dichlorofluorescin diacetate (DCFH‐DA) in FPLG‐treated foam cells declined by nearly 20 times compared with the LPS and ox‐LDL treated group, showing the superior ROS‐scavenging ability of FPLG NPs (Figure 2D,E). Consistently, a slump of the DCFH‐DA fluorescence in the FPLG‐treated group was also detected by fluorescence microscope, which further validated the anti‐ROS capacity of FPLG NPs (Figure 2K).

Apart from ROS overload, the accumulation of lipid peroxide is another major factor that leads to macrophage ferroptosis. Dysregulated cholesterol (CHO) metabolism in foam cells, characterized by elevated ox‐LDL uptake, excessive lipid accumulation and impaired CHO efflux, provides substrates for lipid peroxidation and serves as the culprit of ferroptosis. To determine how the PDA‐oriented NPs were involved in the lipid metabolism of foam cells, we conducted a flow cytometry study to quantify the lipid uptake, accumulation and efflux of these nanoparticles‐treated foam cells. Figure 2F,G show that treatment with PDA‐PEG suppresses cellular uptake of Dil‐labeled ox‐LDL by around 68%, and coincubation with PLG and FPLG downregulates lipid uptake to a level almost the same as the control group. BODIPY is frequently used to probe intracellular lipid droplets, and Figure 2H,I exhibits a steep decline of lipid content within foam cells treated with PDA NPs. PDA‐PEG treatment decreased lipid accumulation by about 22.7%, while PLG and FPLG treatment almost eliminated excessive lipid in macrophages. Moreover, Oil Red O (ORO) staining was conducted to assess the lipid efflux of foam cells. As shown in Figure 2L, coincubation with PDA‐PEG, PLG, and FPLG NPs all efficiently enhance reversed cholesterol transport in foam cells which is manifested by a sharp decrease in ORO+ areas. Altogether, these results demonstrated the extraordinary capability of FPLG NPs to dynamically regulate intracellular lipid metabolism in a synergetic manner.

Acting as a critical endogenous antioxidant system, nuclear factor erythroid 2‐related factor 2 (NRF2) and glutathione peroxidase 4 (GPX4) coordinate with each other to form a vital defense against lipid peroxidation and ferroptosis. Dysfunction of the GPX4‐NRF2 antioxidant mechanism in foam cells exacerbates oxidative stress, leading to fatal lipid peroxidation. In addition, long‐chain acyl‐CoA synthetase 4 (ACSL4) is another critical enzyme that drives ferroptosis as it activates peroxidization of polyunsaturated fatty acids.^[^ ^27^ ^]^ Thus, we further studied whether PDA NPs could regulate the expression of GPX4, NRF2, and ACSL4 in foam cells. After coincubation with various PDA NPs for 12 h, these foam cells were collected and the western blotting experiment was conducted to quantify the level of NRF2, GPX4, and ACSL4. Representative images are shown in Figure 2J, where a significant upregulation of NRF2 and GPX4 in PLG and FPLG‐treated groups can be observed. Accordingly, the intracellular ACSL4 level exhibited an obvious reduction following FPLG treatment, as shown in Figure 2J. Further analysis revealed that coculture with FPLG NPs increased NRF2 and GPX4 levels by around 2.6‐fold and threefold and inhibited ACSL4 expression by about 2.9‐fold (Figure 2M–O). Figure 2P demonstrates that the total iron concentration of the FPLG group was drastically reduced (≈45%), which favored the downregulation of ferroptosis induced by FPLG NPs.Collectively, these in vitro experiments proved that the PDA NPs, particularly FPLG NPs, boasted excellent abilities to eliminate excessive ROS, regulate lipid metabolism and recover impaired antioxidant signaling. This “three in one” effect synergizes to counteract with the overactivated ferroptosis in foam cells, resolving inflammation in the microenvironment and thus leading to plaque regression.

### 2.4 Targeting Efficacy and Biodistribution of FPLG NPs

**Figure 3:**
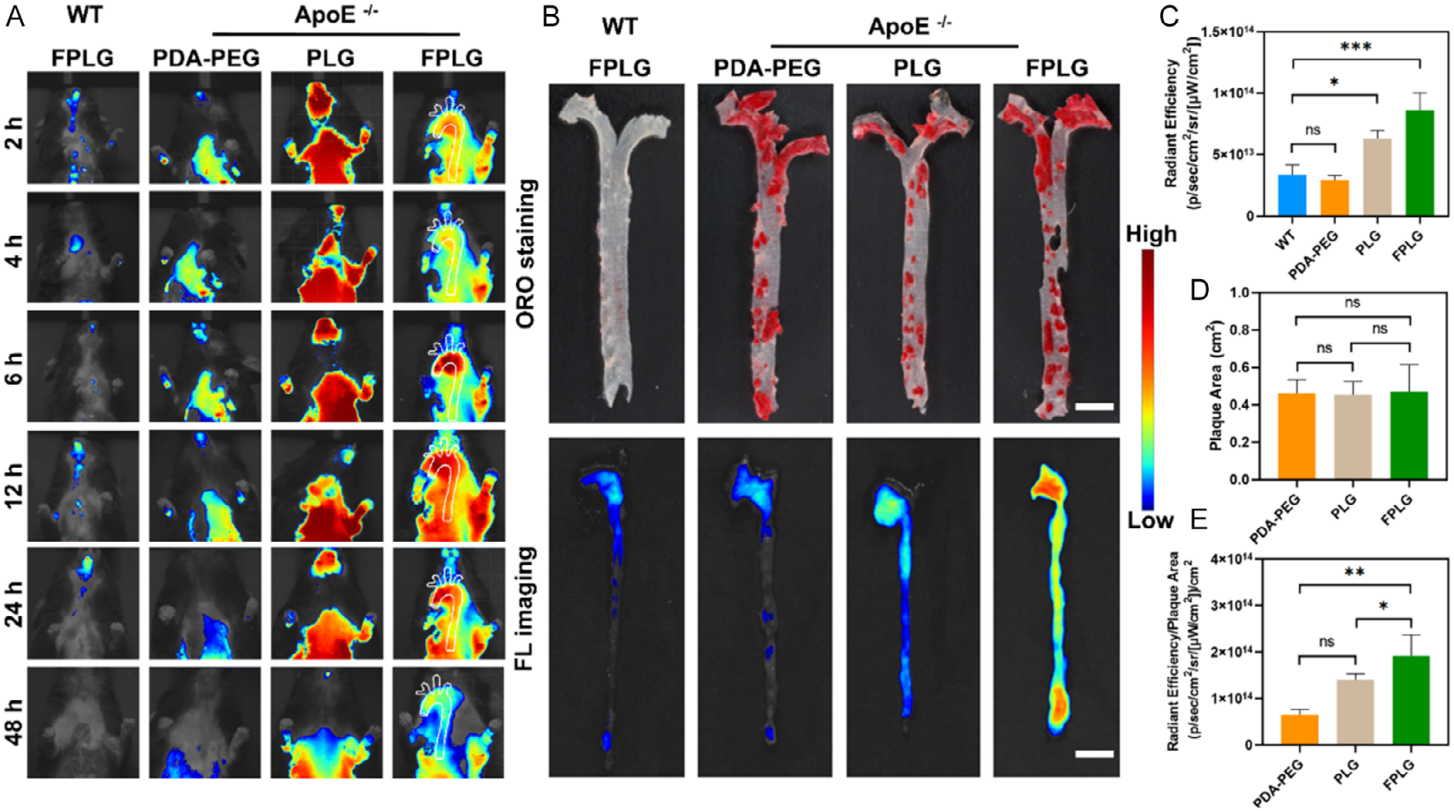
In vivo targeting efficiency of the FPLG NPs. A) In vivo fluorescence images of AS and WT mice treated with different PDA NPs intravenously at the timepoint of 2, 4, 6, 12, 24, and 48 h post injection. B) Ex vivo fluorescence images and the corresponding ORO staining images of the aortas harvested from AS and WT mice 48 h after PDA NP administration. Scale bar: 5 mm. C) Quantitative results of the radiant efficiency of the aortas in ex vivo images (*n* = 3). D) Quantitative results of the plaque area in the AS mice (*n* = 3). E) Quantitative results of the radiant efficiency divided by the plaque area of the same AS mice (*n* = 3). Data are expressed as mean  ± SD. ns, no significance, *p < 0.05; **p < 0.01; ***p < 0.001, and ****p < 0.0001.

Previous studies reported that foam cells within atherosclerotic plaques, which are particularly prone to ferroptosis, canonically express folate receptors at a high level, making folic acid surface modification a promising strategy for our PDA NPs to specifically target vulnerable plaques.^[^ ^16^, ^28^ ^]^ To investigate the in vivo targeting efficacy and biodistribution of these PDA NPs, we created the atherosclerotic mouse model and labeled the PDA NPs with the near‐infrared (NIR) fluorescent dye IR820 which was then administrated to AS mice and C57BL/6 mice (wild‐type, WT) intravenously. The atherosclerotic mouse model was constructed by feeding the ApoE^−/−^ mice with a high‐fat diet continuously for 12 weeks, and ORO staining of mouse aortas confirmed the formation of AS plaques (**Figure** **3**B). The AS mice were randomly divided into three groups, namely PDA‐PEG, PLG and FPLG group. A group of chow‐fed WT mice were also injected with fluorescent FPLG NPs through the tail vein as a control group. At the timepoint of 2, 4, 6, 12, 24, and 48 h post injection, in vivo imaging was performed on these mice. Then, these mice were sacrificed immediately, and the aortas and major organs were harvested for ex vivo imaging.

According to the in vivo images displayed in Figure 3A, at 6 h post intravenous injection of PDA NPs, there were tangible florescent signals in the aortas of the AS mice, the intensity of which was significantly higher than that of the WT mice. The strongest fluorescent signal of the PDA NPs accumulated within AS plaques emerged at the timepoint of 12 and 24 h postinjection, and from then on, the NIR fluorescence in the aortas faded gradually. Among all the PDA NPs, the FPLG NPs exhibited the most outstanding targeting efficiency to atherosclerotic plaques as the fluorescent intensity of the FPLG group was higher than other groups. To further verify whether the PDA NPs specifically accumulated in the lesion sites, we executed ex vivo fluorescent imaging and ORO staining of the aortas. As shown in Figure 3B and Figure 3D, there was no significance in the plaque area of all the AS mice, but the fluorescent intensity between groups varied greatly. The FPLG groups yielded the highest radiant efficiency 48 h postinjection which was about 2.5 folds of the PDA‐PEG group and 1.4 folds of the PLG group, indicating the promoted targeting efficacy due to the surface modification (Figure 3C). Moreover, the corresponding ORO staining images of the aortas showed a high relevance between NP accumulation and plaque existence, with FPLG groups possessing the peaked value of radiant efficiency/plaque area (Figure 3E). Taken together, these results demonstrated that our nanosized PDA materials effectively accumulated in the plaques due to the EPR effect,^[^ ^29^ ^]^ and the surface functionalization of LA and folic acid improved their targeting efficacy even more. Immunofluorescence of the aortas from FPLG NP‐treated mice presented the co‐localization between FPLG NPs and CD68, providing further evidence for the foam cell‐targeting ability of FPLG NPs (Figure. S6, Supporting Information). As mentioned above, folic acid modification navigated FPLG NPs to foam cells within atherosclerotic plaques which are featured by elevated expression of folate receptors, and LA further reinforced its permeability and facilitated its accumulation in plaque areas.^[^ ^14^ ^]^

We also investigated the biodistribution of these PDA NPs through ex vivo imaging of major organs harvested from AS mice. Figure S7, Supporting Information demonstrates that these PDA NPs preferentially accumulate in the liver and kidney rather than the heart and spleen after 48 h of internal metabolism. These data showed that PDA NPs were mainly cleared through the liver and kidney.

### 2.5 PET/CT Imaging of ^68^Ga‐FPLG NPs in as Mice

**Figure 4:**
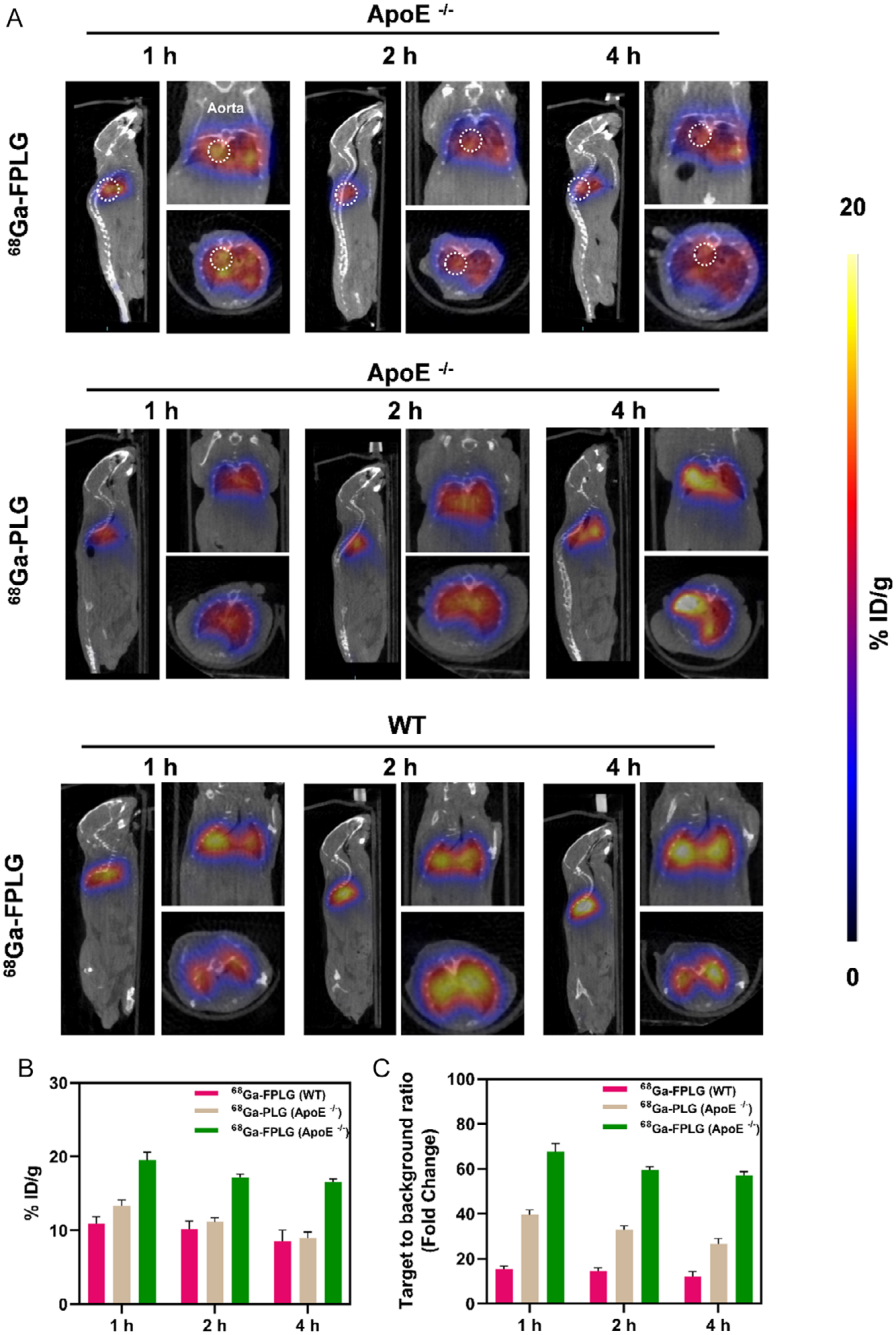
PET/CT imaging demonstrated the intraplaque accumulation of 68Ga‐FPLG NPs. A) Representative PET/CT images of ApoE^−/−^ and WT mice at different time points postinjection of ^68^Ga‐FPLG NPs or ^68^Ga‐PLG NPs. The white cycle highlights the diseased aortas of the AS mice. B) ROI quantification of aortas from imaging in panel A (*n* = 3). C) Ratios trend of aorta‐to‐muscle were computed in each model from 1 to 4 h (*n* = 3).

Encouraged by the prominent targeting efficiency of FPLG proved by fluorescent imaging, we further explored the diagnostic potential of 68Ga‐FPLG NPs using in vivo PET/CT imaging. We conducted longitudinal PET/CT imaging in APOE^−/−^ and WT mice following intravenous administration of ^68^Ga‐FPLG or ^68^Ga‐PLG. Time‐activity analyses at 1, 2, and 4 h post‐injection revealed optimal contrast at the 4‐hour timepoint. Quantitative comparison in APOE^−/−^ mice demonstrated significantly higher aortic accumulation of ^68^Ga‐FPLG (%ID/g = 16.5 ± 0.4) compared to the non‐targeted ^68^Ga‐PLG (%ID/g = 9 ± 0.5) (**Figure** **4**A–C). This targeting specificity was further confirmed by aortic‐to‐muscle uptake ratios, with ^68^Ga‐FPLG showing a 2.14‐fold improvement in the target‐to‐background ratio (TBR = 57.09 ± 1.27 vs 26.63 ± 2.11 for ^68^Ga‐PLG). In addition, the absence of significant ^68^Ga‐FPLG accumulation in WT aorta (%ID/g = 8.6 ± 1.3; TBR = 12.13 ±  1.42) correlated with low FR expression in healthy vasculature, confirming pathological plaque‐specific FA receptor engagement (Figure 4A–C). Overall, the targeted delivery and rapid accumulation of ^68^Ga‐FPLG NPs underscore their potential as a precision imaging tool for AS, enabling early and accurate plaque identification and real‐time monitoring of therapeutic interventions.

### 2.6 In Vivo Atherosclerosis Alleviation via FPLG NP Treatment

**Figure 5:**
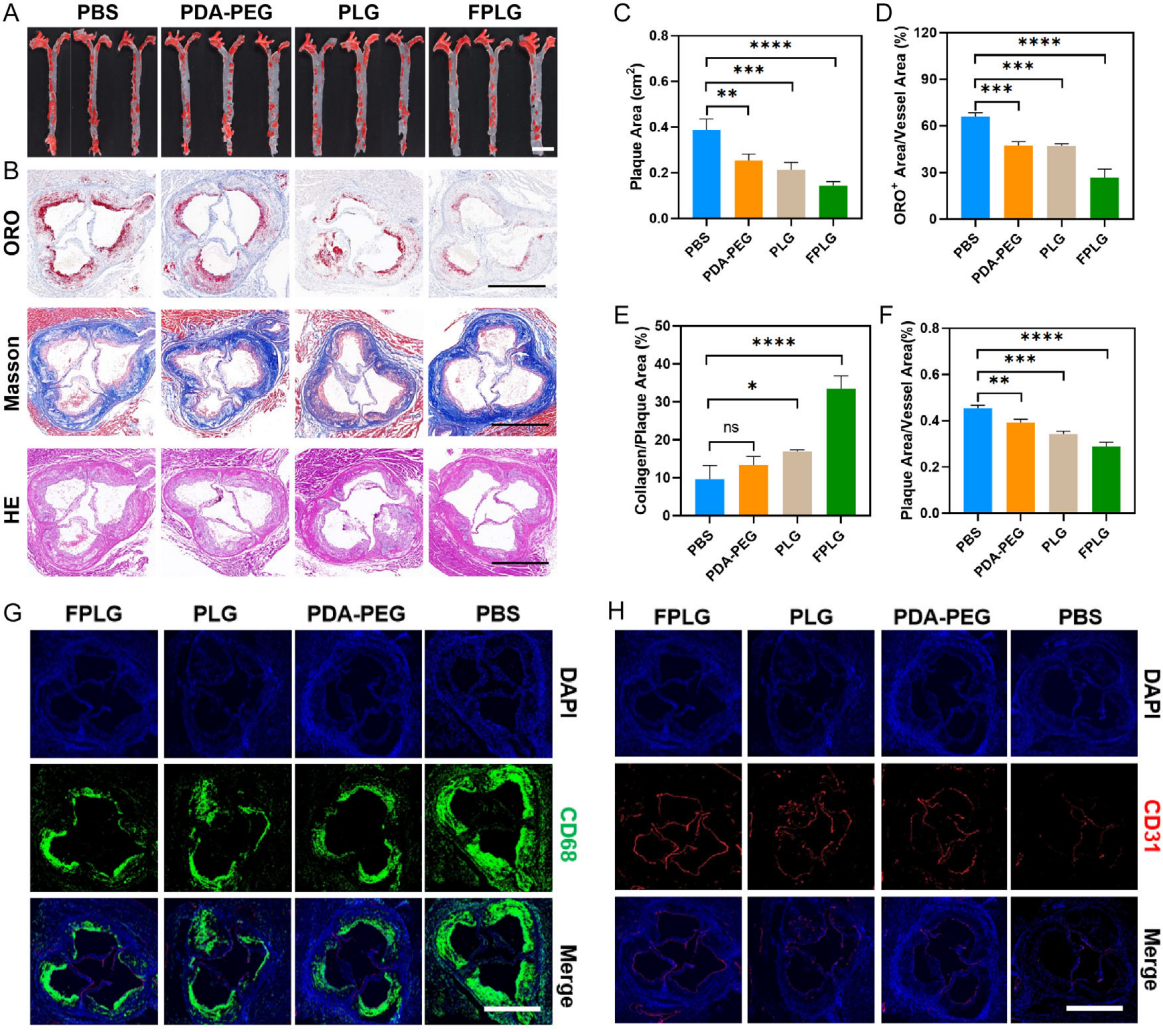
In vivo anti‐AS effects of FPLG NPs. A) Representative en face ORO staining photographs of the aortas collected from AS mice undergoing various treatments. Scale bar: 5 mm, B) representative micrographs of aortic root sections stained with ORO, Masson's trichrome and H&E collected from AS mice receiving various treatments. Scale bar: 200 μm, C) quantitative analysis of the plaque area observed in (A), D) the ratio of ORO+ area to the vessel area, E) the ratio of collagen area to the plaque area and, F) the ratio of plaque area to the vessel area observed in (B) (*n* = 3), G) representative immunofluorescence images of the aortic root section from various groups stained for CD68. Scale bar: 200 μm, H) representative immunofluorescence images of the aortic root section from various groups stained for CD31. Scale bar: 200 μm. Data are expressed as mean ± SD. ns, no significance, *p < 0.05; **p < 0.01; ***p < 0.001, and ****p < 0.0001.

Based on the above results, we next explored the in vivo anti‐AS effect of the PDA NPs. The AS mice were randomly separated into four groups including the PBS, PDA‐PEG, PLG, and FPLG group. Treatment of PBS and different PDA NPs was conducted twice a week at a concentration of 15 mg kg^−1^ for another month, during which time the high‐fat diet was continuously imposed on the ApoE^−/−^ mice. Subsequently, all the mice were euthanized and scarified to evaluate the in vivo antiatherosclerosis efficacy of several PDA NPs. The schematic illustration can be seen in Figure S8, Supporting Information. **Figure** **5**A shows the ORO staining images of the aortas collected from AS mice undergoing various treatments. Both ORO staining images and quantitative analysis revealed a significant decrease in the plaque area after PDA NP treatment, with the FLP NP treatment ameliorating AS progression by about 45.8% and the FPLG group reducing the plaque area by ≈64.5% (Figure 5A and Figure 5C). Meanwhile, sections of aortic roots from AS mice were also obtained for the subsequent histological analysis. Administration of FPLG NPs to AS mice retarded AS development and progression to a considerable extent, as the ORO+ area was reduced by around 61% and the plaque area indicated by the H&E staining was reduced by around 43.5% compared with the PBS group (Figure 5D–F). Surface modification of L‐arginine and folic acid of the PDA NPs remarkably augmented their anti‐AS effects, as PDA‐PEG alone alleviated plaque growth by only 27.6% and PLG NPs by 31.8% (Figure 5D–F), both inferior to the 43.5% reduction induced by FPLG NP treatment. Along with the shrinkage of the lesion area, elevated stability of these atherosclerotic plaques was also confirmed by Masson's trichrome staining which exhibited the increased collagen contents in the FPLG group (Figure 5B). Quantitative analysis displayed in Figure 5E revealed that FPLG NP treatment promoted collagen deposition by about 3.46 folds, which is in line with the data presented in Figure 5B. In addition, FPLG treatment effectively mitigated macrophage infiltration and restored endothelium barrier in the lesion sites as evidenced by the immunofluorescence images displayed in Figure 5G–H. Altogether, these results demonstrated the extraordinary in vivo anti‐AS activity of PDA NPs, especially the FPLG NPs.

### 2.7 In Vivo Antiferroptosis Performance of FPLG NPs

**Figure 6:**
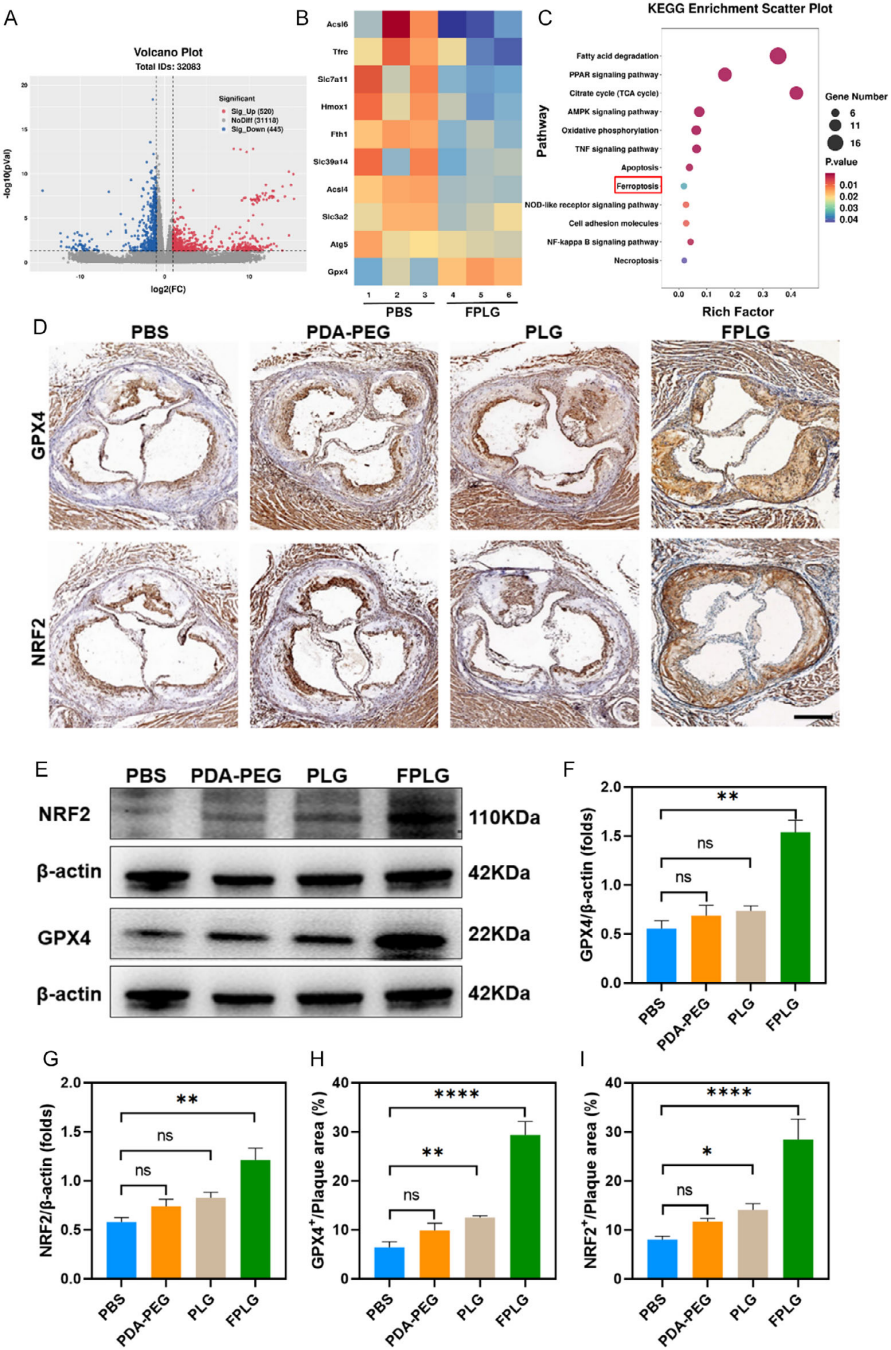
In vivo antiferroptosis mechanism and efficacy of FPLG NPs. A) Volcano maps of upregulated and downregulated genes in aortas after PBS versus FPLG treatments. Cutoffs were set at |log2(FC)| > 2.0, *p* < 0.05. B) Heatmap of the differentially expressed genes highly associated with ferroptosis within aortas. C) Bubble diagram of KEGG enrichment analysis from DEGs in PBS groups compared to the FPLG treated groups. D) Representative immunohistochemical images of aortic root sections stained for GPX4 and NRF2 obtained from AS mice of different groups. Scale bar: 100 μm. Quantitative analysis is shown in F,G) (*n* = 3). E) Representative western blotting bands of the NRF2 and GPX4 within the aortas collected from AS mice following various treatments, with the corresponding quantification displayed in H,I) (*n* = 3). Data are expressed as mean ± SD. ns, no significance, *p < 0.05; **p < 0.01; ***p < 0.001, and ****p < 0.0001.

In order to further elucidate the underlying mechanism of the in vivo therapeutic effects brought about by FPLG NPs, transcriptome sequencing analysis was performed on the aortas isolated from the AS mice of the PBS and FPLG group. The results showed that FPLG treatment led to 965 differentially expressed genes (DEGs) in the aortas, with 520 upregulated and 445 downregulated genes (**Figure** **6**A). Notably, the significant downregulation of ACSL4 curtails polyunsaturated fatty acid‐phospholipid synthesis, directly limiting lipid peroxidation substrates in plaque macrophage ‐ a pivotal trigger of ferroptotic cell death. Concurrently, GPX4 upregulation enhances glutathione‐dependent detoxification of lipid hydroperoxides, converting cytotoxic peroxides into inert alcohols to stabilize cellular membranes (Figure 6B).^[^ ^30^ ^]^ The KEGG functional enrichment analysis showed that FPLG treatment reduced the ferroptosis signature (Figure 6C). Moreover, compared to the PBS group, FPLG treatment also downregulated pathways linked to AS development, including the Peroxisome Proliferators‐activated Receptors (PPAR) signaling pathway, NOD‐like receptor signaling pathway, and apoptosis (Figure 6C). The modulation of the PPAR signaling pathway, particularly PPARγ, highlights the anti‐inflammatory and antiferroptotic roles of FPLG NPs. PPARγ is known to exert protective effects against AS development by regulating lipid metabolism and inflammatory responses.^[^ ^31^ ^]^ At the same time, the suppression of NOD‐like receptor signaling further attenuates NLRP3 inflammasome activation, breaking the vicious cycle between ferroptosis‐derived DAMPs, and inflammation. By simultaneously targeting lipid peroxidation cascades (ACSL4↓/GPX4↑), metabolic dysregulation (PPARγ↑), and inflammatory amplification (NLRP3↓), FPLG NPs reshape plaque microenvironment homeostasis, demonstrating therapeutic potential for AS.

In line with the above results, an increased level of GPX4 and NRF2 in the aortas of AS mice after FPLG NP treatment was also verified by immunohistochemistry analysis and western blotting results. As mentioned above, GPX4 and NRF2, two critical regulators of intracellular oxidative stress, usually negatively modulate the level of macrophage ferroptosis. In Figure 6D, representative micrographs of immunohistochemical staining for GPX4 and NRF2 are shown. The upregulated level of GPX4 and NRF2 in the aortic root induced by FPLG NP treatment can be easily observed from Figure 6D. Quantitative analysis in Figure 6H–I demonstrated that the GPX4 level ramped up by almost fourfolds and the NRF2 level by threefolds following FPLG NP treatment. Intriguingly, PDA‐PEG or PLG NPs failed to restore GPX4 expression in atherosclerotic plaques as compared with FPLG NPs, which may be partly due to the outstanding targeting capacity to foam cells benefiting from folic acid modification. Results from western blotting assays were in concert with the above findings, with the GPX4 level of the aorta increased by 2.6 folds and NRF2 level by 3.3 folds due to FPLG NP treatment (Figure 6E–G). Collectively, these results confirmed that FPLG restrained AS progression via upregulation of GPX4 and NRF2 which effectively curbed macrophage ferroptosis within the plaque.

### 2.8 In Vivo Biocompatibility Assessment of FPLG NPs

When the AS mice were under treatment of the diverse PDA NPs, their body weight was examined at regular intervals. No distinct weight loss was observed during the treatment, suggesting the low biotoxicity of PDA NPs at least on a therapeutic dose (15 mg kg^−1^) (Figure. S9A, Supporting Information). At the endpoint of the treatment, major organs including the heart, liver, spleen, lung, and kidney of these mice were harvested. Histological analysis of the corresponding sections revealed negligible pathological consequences of PDA NP treatment as shown in Figure S9D, Supporting Information indicating that the PDA NPs have high biological availability. Moreover, we conducted biochemistry tests on the blood samples collected from the AS mice and found that treatment by FPLG NPs slightly reduced the CHO level in the serum. However, the levels of triglyceride (TG) and LDL did not show a significant change after FPLG treatment, which indicates that FPLG NPs do not work in a lipid‐lower‐dependent manner (Figure. S9B, Supporting Information). The levels of alanine transaminase (ALT), blood urea nitrogen (BUN), creatinine (CREA), and uric acid (UA) were also evaluated to assess whether there existed liver or kidney dysfunction. Consistent with the results of the histologic analysis, Figure. S9C, Supporting Information demonstrates that these serum biochemical indicators all remain within the normal range. To sum up, the above results provided convincing evidence about the in vivo biosafety of these PDA NPs.

## 3 Conclusion

Overall, we successfully engineered a folate and LA functionalized polydopamine nanoplatform (FPLG NPs) to address foam cell ferroptosis and therefore alleviate atherosclerosis. The synergistic design of FPLG NPs combines antioxidative PDA, arginine‐enhanced radical scavenging, and folate‐mediated targeting to foam cells, which ultimately achieved encouraging theranostic effects. In vitro and in vivo fluorescence both visualized the superior accumulation of FPLG NPs in foam cells and AS plaques, denoting the excellent targeting efficacy of this nanomaterial. Specially, PET/CT imaging enabled by ^68^Ga‐chelation was introduced in a bid to further investigate the in vivo targeting efficiency and pharmacokinetics of the FPLG NPs. Results from PET/CT imaging highly correlated with the findings of in vivo fluorescence, demonstrating preferential accumulation of FPLG NPs in ferroptosis‐enriched atherosclerotic plaques. Subsequently, the FPLG NPs induced a pronounced decline of macrophage ferroptosis via eliminating ROS, inhibiting ACSL4 expression, and reducing iron ion content, which further regulate lipid metabolism as well as restoring endogenous antioxidant defense (GPX4 and NRF2). Administration of FPLG NPs to AS mice effectively induced plaque regression by up to 60%, which was accompanied by a prominent downregulation of foam cell ferroptosis in the lesion site. Neither significant weight loss nor pathological changes of major organs were observed throughout the treatment, suggesting the high biocompatibility of the FPLG NPs. Altogether, these results highlight FPLG NPs as a novel ferroptosis‐targeted theranostic strategy for atherosclerosis management. Future work should explore clinical translation, long‐term safety, and combinatorial therapies leveraging this platform.

## 4 Experimental Section

4.1

4.1.1

##### Materials

Dopamine hydrochloride (C_8_H_11_NO_2_.HCl) and L‐Arginine (LA, C6H14N4O2) were purchased from Aladdin (China). Sodium hydroxide (96≥%, NaOH) and NH4OH solution (28 wt%) were obtained from Nanjing Reagent in China. Amine‐PEG2000‐amine (NH_2_‐PEG2000‐NH_2_) was obtained from Ponsure Biotechnology. FA‐modified PEG2000‐amine (FA‐PEG2000‐NH_2_) was obtained from Xi'an Ruixi Biological Technology Co. Ltd, China. A CCK8 was from Beyotime, China. Human oxidized low‐density lipoprotein was purchased from YEASEN, China. LPS was purchased from Sigma (USA).

##### Preparation of PDA, PDA‐PEG, PLG, and FPLG Nanoparticles

Polydopamine (PDA) nanoparticles were synthesized through oxidative self‐polymerization following an adapted literature protocol.^[^ ^32^ ^]^ Briefly, 100 mg of dopamine hydrochloride (Sigma‐Aldrich) was dissolved in 0.8 mL of aqueous ammonia solution under vigorous magnetic stirring for 10 min to ensure complete dissolution. Subsequently, 2 mL of ethanol (99.8%, Aladdin) was introduced into the reaction mixture. The polymerization reaction was allowed to proceed under ambient conditions for 12 h with continuous stirring, resulting in the formation of a characteristic black colloidal suspension. The crude product was subjected to purification through three times centrifugation followed by redispersion in deionized water to remove unreacted monomers and oligomeric byproducts. The final purified PDA NPs were lyophilized for 48 h to obtain a stable powder for subsequent characterization and quantification. The PLG or FPLG NPs were synthesized by dropping 5 mL of PDA aqueous solution (1 mg/mL, pH = 9 adjusted by NH_4_OH solution) into the reaction bottle containing 10 mg LA and stirring for 24 h.^[^ ^19^ ^]^ The obtained PLG was purified by centrifugated under 15 000 rpm for 30 min and washed three times with water. The FPLG NPs were synthesized by adding 5 mg PLG to 50 mg NH2‐PEG2000‐NH2 or FA‐PEG2000‐NH2 in 5 mL DI water (pH = 8.5). After 24 h stirring, the FPLG was obtained and washed with DI water for 3 t.^[^ ^7^ ^]^

##### Radiolabeling of FPLG NPs

The radiolabeling procedure was performed by chelating ^68^Ga^3+^ (3 mCi in 4 mL) with FPLG NPs (2 mL, 1 mg mL^−1^) under optimized pH conditions (5–6) maintained through titration with 1 M sodium acetate buffer. The reaction system was maintained at 60 °C with continuous agitation for 30 min to facilitate chelation. Free ^68^Ga^3+^ were removed through centrifugal ultrafiltration (30 kDa MWCO) with successive aqueous rinses until filtrate radioactivity reached background levels. Quantitative assessment of labeling efficiency was conducted by gamma‐counting measurements, calculated as: the radioactivity in the ultrafiltration tube/(the radioactivity in the ultrafiltration tube+ the radioactivity in the filtration solution).

##### Cell Culture

The RAW 264.7 was obtained from Procell, China. PBS, DMEM, fetal bovine serum (FBS), and penicillin/streptomycin were purchased from Gibco. All cells were cultured according to the guidelines in appropriate media supplemented with 10% FBS and 1% penicillin/streptomycin in an incubator at 37 °C, 5% CO_2_% and 95% relative humidity.

##### Cellular Cytotoxicity

In vitro cytotoxicity of PEG‐PDA, PLG NPs, and FPLG NPs toward RAW 264.7 cells, SMCs and MAECs was assessed via the CCK‐8 assay. In brief, cells were seeded in 96‐well plates at a density of 10 000 cells per well and cultured overnight. Following this, cells were treated with a medium containing various concentrations of nanoparticles (0, 20, 40, 60, 80, and 100 μg mL^−1^) for 24 h. Subsequently, cell viability was quantified according to the standard CCK‐8 assay protocol.

##### Cellular Uptake

Foam cells were used to assess cellular uptake. RAW 264.7 cells were seeded in 6‐well plates and cultured overnight. The media were then replaced with fresh media containing 80 μg mL^−1^ ox‐LDL and 200 ng/mL LPS to create the foam cell model. After 24 h, cells were treated with PDA‐PEG, PLG, and FPLG NPs (100 μg mL^−1^) for another 12 h. Then the cells were collected and analyzed via a flow cytometer (Beckman Colter, USA) in the APC channel. For qualitative evaluation, cells were seeded in 20 mm confocal dishes (Biosharp) and treated similarly. Finally, images were captured using a laser confocal microscope (Leica, Germany).

##### ROS Scavenging Ability

The in vitro ROS scavenging abilities of PEG‐PDA, PLG NPs, and FPLG NPs were assessed using the standard DCFH‐DA assay. Briefly, RAW 264.7 cells were seeded in 6‐well plates at 5 × 10^5^ cells/mL and cultured for 24 h. For the positive control groups, the medium was replaced with fresh medium containing LPS (200 ng/mL). For the experimental groups, the medium was replaced with fresh medium containing both LPS (200 ng/mL) and different nanoparticles (PEG‐PDA NPs, PLG NPs, and FPLG NPs, 100 μg/mL). After 24 h of incubation, the cells were stained with the DCFH‐DA probe for flow cytometry analysis. For CLMS detection, cells were cultured in Confocal dishes (35 mm, NEST) and treated as described above.

##### In vitro *lipid regulation analyses*

ORO staining was used to assess the inhibitory effects of different nanoparticles on foam cell formation. RAW 264.7 cells were cultured in 6‐well plates at a density of 5×10^5^ mL for 24 h. The positive control group was treated with a medium containing LPS (200 ng/mL) and ox ‐ LDL (80 μg/mL). Other groups were treated with medium containing LPS (200 ng/mL), ox ‐ LDL (80 μg/mL), and different nanoparticles (PDA‐PEG, PLG, and FPLG NPs, 100 μg/mL). After another 24 h, cells were washed, fixed, and stained with ORO (sigma) for 30 min and observed under a microscope.

##### Establishment of Atherosclerosis Model

Female C57BL/6 mice (as healthy controls) and ApoE ‐/‐ mice (for the atherosclerosis model) were obtained from China's GemPharmatech Company. All animal procedures were authorized by the Institutional Animal Care and Use Committee of Jinling Hospital. The atherosclerosis model was developed by prior literature.^[^ ^14^ ^]^ In brief, 6‐week‐old ApoE ‐/‐ mice were given a high‐fat diet (XT108C, XIETONGSHENGWU, China) for 12 weeks to establish the atherosclerotic plaque, verified via ORO staining.

##### In vivo *and* ex vivo *NIRF imaging and biodistribution assays*

ApoE^−/−^ and WT mice were intravenously administrated IR820‐labeled PDA‐PEG, PLG, and FPLG NPs (15 mg/kg). Whole‐body fluorescence imaging was performed at 2, 4, 8, 12, 24, and 48 h postinjection via an IVIS Spectrum imaging platform. After 48 h, euthanized mice underwent systematic collection of major organs (aorta, heart, liver, spleen, lungs, and kidneys) to allow ex vivo fluorescence‐based quantification of nanoparticle bio‐distribution.

##### Micro‐PET/CT imaging

AS and WT mice were administered intravenous injections of 50 μCi ^68^Ga‐FPLG. Static PET/CT scans were then performed at 60, 120, and 240 min postinjection to obtain images. Regions of interest (ROI) were delineated on whole‐body axial images of the aorta. Muscle tissue was selected as the background, and the percentage of injected dose per gram of tissue (%ID/g) was determined at multiple time points.

##### Treatment of AS mice

The established AS mice were randomly divided into four groups and treated with (a) PBS, (b) PDA‐PEG, (c) PLG NPs, and (d) FPLG NPs, respectively. All the solutions were injected via intravenous with a dose of 15 mg/kg. Treatment was given every three days, during which the body weight of mice was recorded. After eight treatments, the mice were euthanized, and their aortas, major organs, and blood samples were collected for subsequent analysis.

##### In vivo *anti‐AS efficacy, mechanisms, and biosafety analysis*

Following different treatments, the whole aortas from selected mice were fixed in 4% paraformaldehyde and subjected to en face Oil Red O staining. The other aortas were fixed and sectioned for Hematoxylin and Eosin (HE), Oil Red O, and Masson staining. Some of the remaining sections were incubated with anti‐GPX4 and anti‐NRF2 antibodies for immunohistochemistry. Immunofluorescent staining for CD68 and CD31 was also performed on other aorta sections. The major organs (heart, liver, spleen, lung, and kidney) were fixed and stained with HE staining. Quantitative analysis was done using ImageJ software. Moreover, serum samples from each mouse were collected to measure AS‐related biochemical indicators LDL, high‐density lipoprotein (HDL), CHO, TG, and biosafety‐related parameters (ALT, BUN, CREA, and UA).

##### Western blot analysis

Whole‐cell protein extraction and following standard western blotting were conducted as previously described.^[^ ^33^ ^]^ In brief, RAW 264.7 cells and the aortas from mice after various treatments were lysed with radio‐immunoprecipitation assay (Beyotime). Total protein concentration was measured by BCA assay. Proteins of equal concentration were separated on 4–12% Bis‐Tris gels (Genscript, China) and transferred to 0.22 μm PVDF membranes (Biorad). The membranes were probed overnight at 4 °C with primary antibodies (anti‐GPX4 and anti‐NRF2 from Abcam), then incubated with horseradish peroxidase‐conjugated secondary antibody (1:5000) for 2 h. Target proteins were detected using a chemiluminescence imaging system (Tanon, China), and band intensity was quantified with ImageJ software.

##### RNA sequencing and bioinformatic analysis

Aortas from mice receiving terminal PBS or FPLG treatments (*n* = 3/group) were harvested for transcriptomic profiling. Total RNA isolation was performed using Trizol reagent (Invitrogen), followed by RNA sequencing and bioinformatic analysis conducted by LC‐Bio Technology (Hangzhou, China).

##### Statistical Analysis

All the quantitative data were presented as the mean ± standard deviation (SD). Statistical analysis was conducted by GraphPad Prism 9.0. An unpaired *t*‐test was used to determine the significant statistical difference in different groups. The data were described according to the *p* values and denoted by (*) for *p* < 0.05, (**) for *p* < 0.01, (***) for *p* < 0.001, and (****) for *p* < 0.0001. N.S. was indicated as not significant.

## Conflict of Interest

The authors declare no conflict of interest.

## Ethics Statement

All animal procedures were approved by the Institutional Animal Care and Use Committee of Jinling Hospital (approval number: DZGDWLS202503000246).

## Supporting information

Supplementary Material

## Data Availability

Research data are not shared.
