# Supplementary material for: Positron Emission Tomography/Computed Tomography Imaging‐Guided Polydopamine Nanoparticles Attenuate Foam Cell Ferroptosis for Targeted Antiatherosclerotic Therapy
Source: Small Sci. 2025 Jul 6;5(9):2500221. doi: 10.1002/smsc.202500221 (PMC12412604; doi:10.1002/smsc.202500221)
Supplement: Supplementary file 1 — Supplementary Material [file SMSC-5-2500221-s001.pdf]

Supporting information

PET/CT imaging-guided polydopamine nanoparticles attenuate foam cell ferroptosis for targeted anti-atherosclerotic therapy

Ximei Dai, Zhiyue Wang, Jiaqi Lu, Yutong Xu, Xingji Liu, Jianchen Qi, Tao Zheng, Feng Wang, Guangming Lu, Longjiang Zhang, Jie Sheng,* and Guifen Yang**

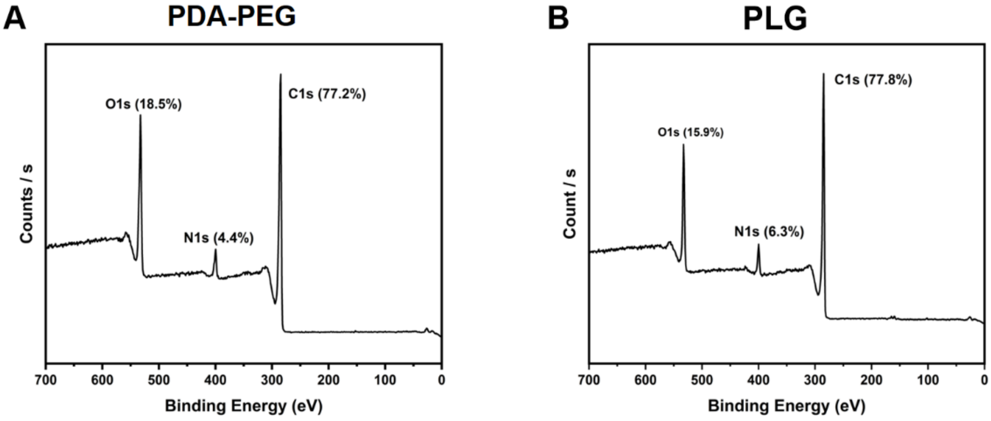

Figure S1. XPS survey scan spectra of PDA-PEG and PLG NPs

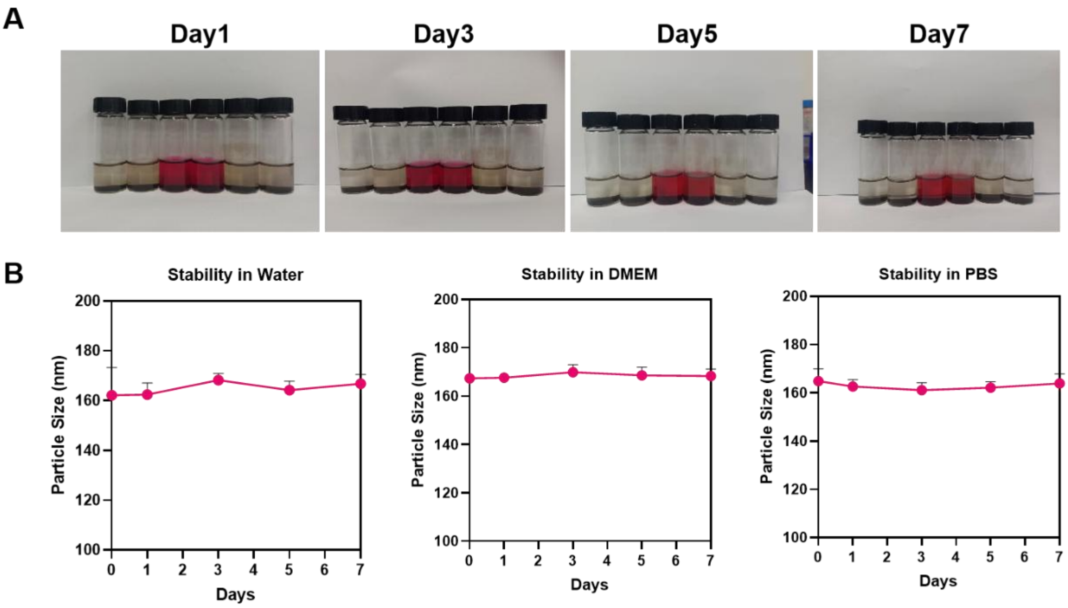

Figure S2. (A) Photographs of FPLG NPs dispersed in water, DMEM medium, and PBS over a 7-day period. (B) Hydrodynamic size measurements of FPLG NPs in

these media during day 1 to day 7

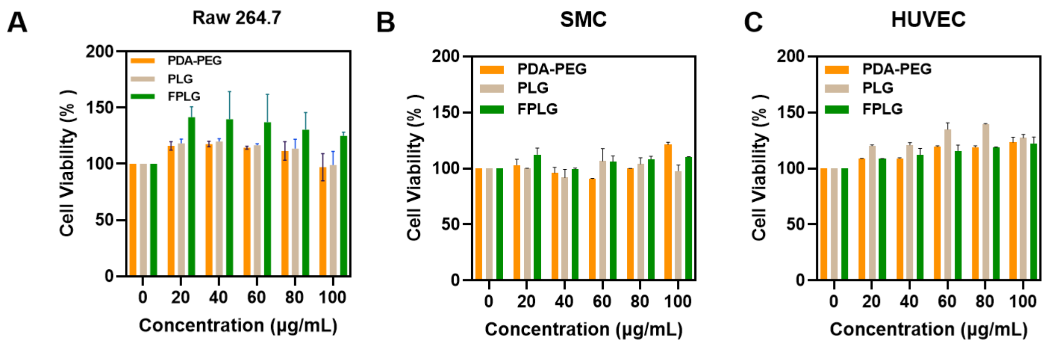

Figure S3. Cell viability of RAW 264.7 cells, SMCs and MAECs treated by PDA NPs at various concentrations tested via CCK-8 assay.

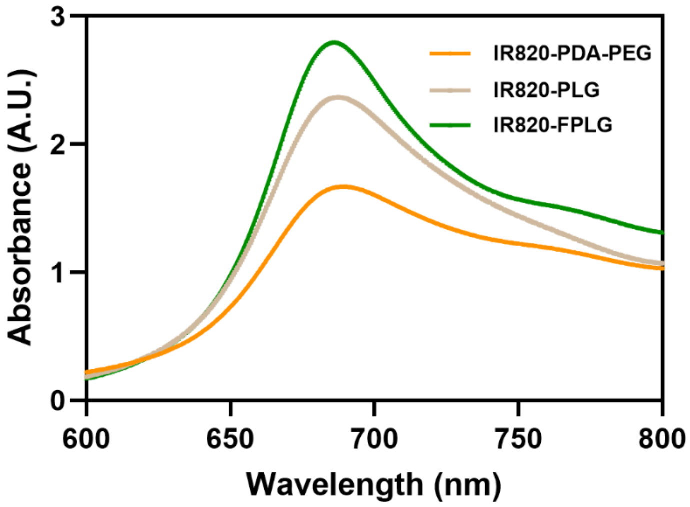

Figure S4. The UV-VIS spectra of IR820 labeled PDA-PEG, PLG and FPLG NPs

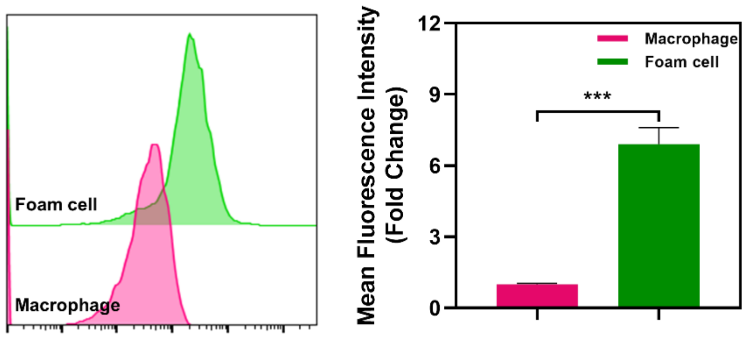

Figure S5. Flow cytometry analysis of foam cells and Raw 264.7 incubated with IR-820 labelled FPLG, with the according mean fluorescence intensity of each group (n=3).

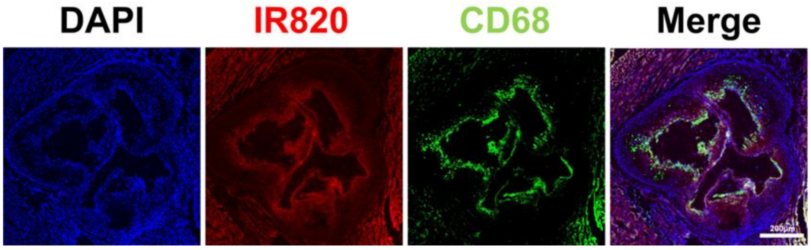

Figure S6. Representative immunofluorescent images of the CD68-stained aortic sections collected from AS mice after intravenous injection of IR820-labelled FPLG NPs. Scale bar: 200μm.

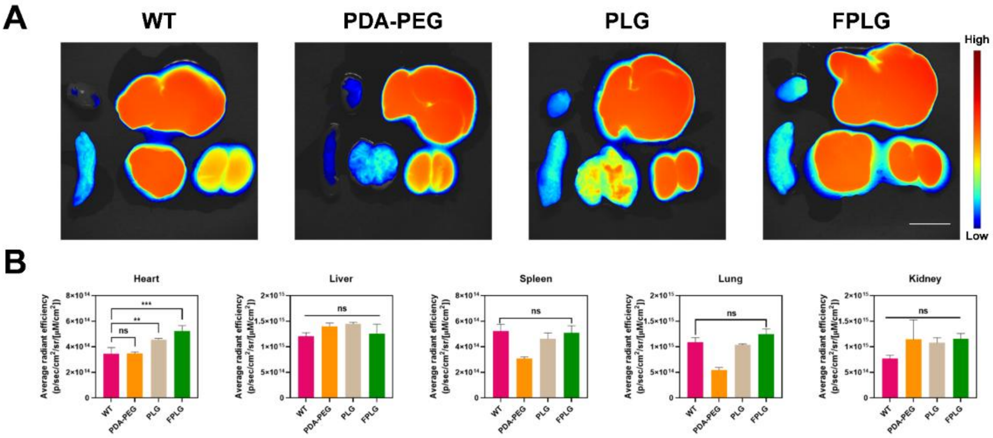

Figure S7. (A) Representative ex vivo fluorescence images of the major organs harvested from the AS mice intravenously injected with IR820-FPLG NPs after 48 hours. Quantitative analysis of the average radiant efficiency are shown in (B). (n=3). Data are expressed as mean ± SD. ns, no significance, *p < 0.05; **p < 0.01; ***p < 0.001, ****p < 0.0001.

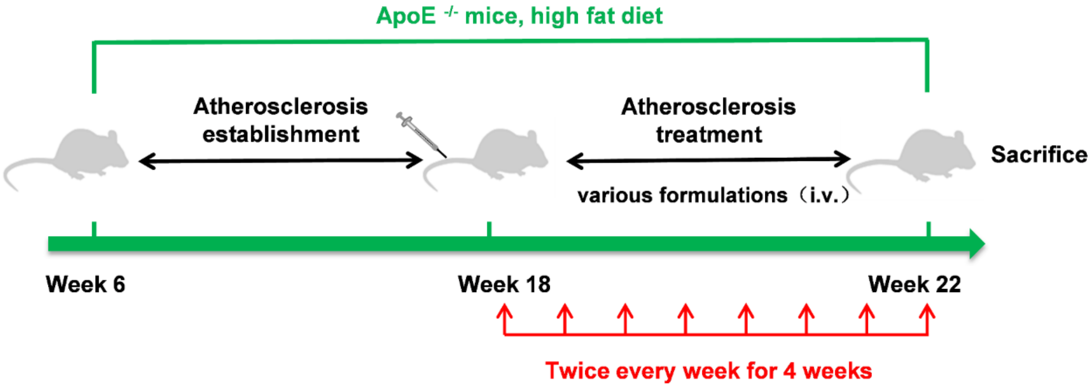

Figure S8. Schematic diagram illustrating the experimental timeline of AS model induction and subsequent therapeutic interventions administered through tail vein

injection, including: PBS, PDA-PEG, PLG and FPLG NPs.

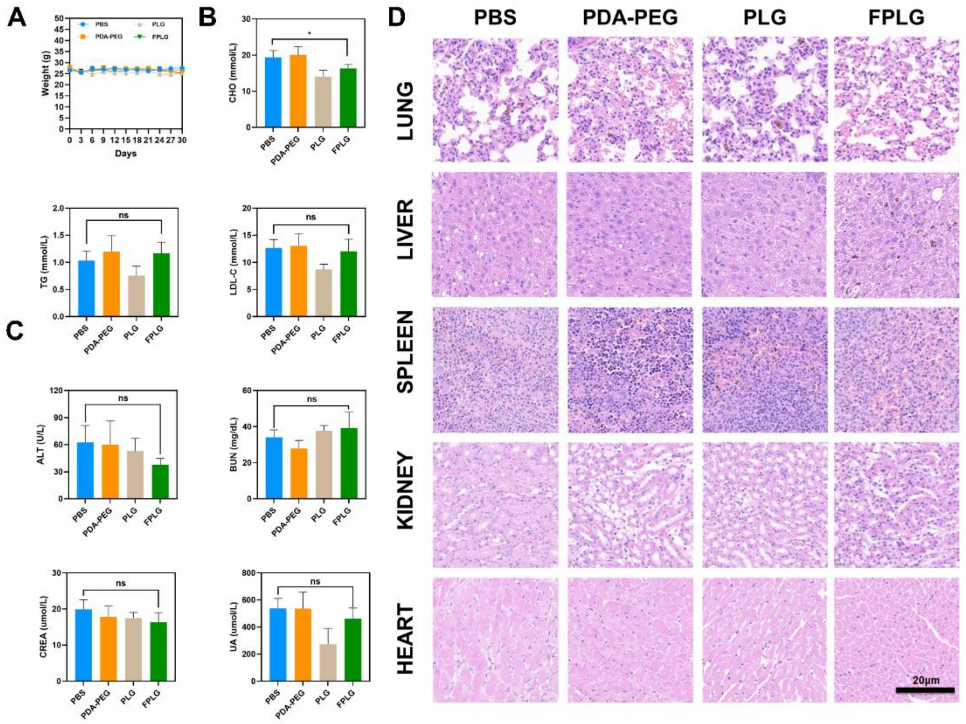

Figure S9. (A) Body weight changes of the AS mice under different treatments (n=8). (B) Levels of the cholesterol (CHO), triglyceride (TG) and low-density-lipoprotein (LDL-C) in the serum of AS mice under various treatments (n=8). (C) Levels of the aminotransferase (ALT), blood urea nitrogen (BUN) and creatinine (CREA) and uric acid (UA) in the serum of AS mice under various treatments (n=8). (D) Sections of major organs attained from AS mice of various groups stained by hematoxylin and eosin (H&E). Scale bar: 20 μm. Data are expressed as mean ± SD. ns, no significance, $*p < 0.05$; $**p < 0.01$; $***p < 0.001$, $****p < 0.0001$.
